# How sociodemographic and hearing related factors were associated with use of hearing aid in a population-based study: The HUNT Study

**DOI:** 10.1186/s12901-016-0028-2

**Published:** 2016-07-16

**Authors:** Anne-Sofie Helvik, Steinar Krokstad, Kristian Tambs

**Affiliations:** Department of Public Health and General Practice, Faculty of Medicine, Norwegian University of Science and Technology (NTNU), Postboks 8905, 7491 Trondheim, Norway; Ear, Nose and Throat Department, St Olav’s University Hospital, Trondheim, Norway; Norwegian National Advisory Unit for Aging and Health, Tønsberg, Norway; HUNT Research Centre, Department of Public Health and General Practice, Faculty of Medicine, Norwegian University of Science and Technology (NTNU), Levanger, Norway; Levanger Hospital, Nord-Trøndelag Health Trust, Levanger, Norway; Norwegian Institute of Public Health, Division of Mental Health, Oslo, Norway

**Keywords:** Hearing aid, Hearing disability, Hearing impairment, Hearing loss, Socioeconomic status, Gender, Hearing handicap

## Abstract

**Background:**

The purpose was to study hearing aid (HA) use in persons 65 years and older, and to investigate how socioeconomic and hearing related factors were associated to use of HA.

**Method:**

This study included 11,602 persons (65 years and above) from the second Nord-Trøndelag Health Study (HUNT2) and the integrated North-Trøndelag hearing loss study (NTHLS) in 1995–1997. Audiometry was taken of all participants. Missing information about use of HA in possible users of HA existed in data from 1103 (9.5 %) of the participants. Effects of sociodemographic variables, low, medium and high frequency hearing thresholds and being bothered by their hearing were explored in men and women, adjusting the effects for each other. Cross tabulations and logistic regression analyses were used.

**Results:**

In all, 14 % (1472 of 10,499) were users of HA, but 62 % had a mean hearing impairment (HI) based on 0.5, 1, 2, 4 kHz over both ears >25 dB. Use of HA was associated with higher education. Adjusting for all covariates and hearing variables, each 10 dB medium frequency threshold shift increased the chance of HA-use by a factor of two-three in both men and women. Having reported being bothered by hearing loss additionally increased the chance around sevenfold. Low frequency hearing thresholds were not associated with HA-use in women. In men, low frequency hearing thresholds up to 50 dB increased odds for use of HA, but low frequency hearing thresholds ≥ 70 dB decreased odds for use of HA. Men living with a spouse had higher odds for using HA compared to men without a spouse. For women there is no difference between those with and without spouse in use of HA. Men and women without spouse did not differ in their use of HA.

**Conclusions:**

About two third of 65 years and older participants had a HI higher than 25 dB, but only one seventh used HA. Use of HA was associated with higher than basic education. Men without a spouse were less likely to use HA compared to men with a spouse.

## Background

Hearing impairment (HI) in older adults (65 years and above) is one of the most common chronic health conditions in the western world today, and the prevalence of HI increases with age [1]. HI has been found to affect about 1/3 of community dwelling persons between 65 and 74 years and about 2/3 of those 75 years and older have been estimated to suffer from HI [2, 3]. The prevalence of HI is around 90 % in the oldest part of the population (80 years and more) [4]. For the vast majority (90 %) of older adults, the HI is sensorineural and irreversible in nature [5]. The most effective treatment for improving hearing among older adults is hearing aid (HA) [6]. Overall, it is estimated that two thirds of the persons with HI would benefit from using HA [7]. However, it is reported that only about 15–30 % of older adults who might benefit from HA in Scandinavia and UK own HA [1, 6, 8, 9], and they do not necessarily use their HA after having acquired it [9–12]. In Norway, the proportion of community living older adults using HA is not known.

HI creates a significant burden for the persons who suffer from it [5]. It may increase the need for community or family support, which may impact negatively older adults’ independence [13]. Also, HI makes the communication harder not only for the persons affected with the hearing loss, but also for people who communicate with them [5]. For older adults HI may affect social interaction, mental health and subjective well-being [14]. Furthermore, it has been documented that mental health and subjective well-being among persons with HI who do not use HA is less good than among those who do [15–17], and further that mental health and cognitive functioning improve after HA-use is established [18, 19]. Thus, if the proportion of older adults with HI fitting and using HA continue to be limited as the western population ages in the years to come, HI-related difficulties may increase the burden on the public community health care services.

Older adults may have need of audiological rehabilitation, including fitting of HA, but do not seek treatment or do not accept use of HA to improve their hearing. There are barriers for both fitting HA [20–22] and for use of HA [22–24]. The cost of the treatment and of the HA itself may also be a reason for not fitting and using a HA [25]. However, in Norway audiological services are financially supported by the government, which means that the audiological rehabilitation, including HA fitting and equipment, is kept to a low cost for the individual patient [26]. When the data was collected the public refunded all costs of hearing aids below a level of 5400NOK (661 EUR) each, and for more expensive hearing aids, like digital aids, the patients payed the difference, but with a maximum pay of 166EUR [26], corresponding to 0.6 % of the mean income after tax in Norway at that time. It is reported that the initial barrier to uptake a HA is reduced when treatment and HA is state funded, but this funding is not necessarily very important for later use of HA [24].

Use of HA has in most clinical audiological studies been found to be positively associated with older persons’ experience of their hearing loss, i.e. those who experience more severe hearing difficulties and are the most bothered by their loss are more likely to use HA after fitting [22]. Furthermore, use of HA have been found to be associated with increasing degree of hearing loss in some studies [11, 12, 27, 28], but the results from two quite recent review reports indicate that degree of HI is not necessarily associated with use of HA after an audiological HA fitting process [22, 24]. In the same reviews the results on an association between high age and use of HA were mixed [22, 24]. None of the studies exploring the importance of gender, living arrangements and level of education for use of HA in older adults after HA fitting have found these factors to affect the outcome. As far as we know, few have studied the relation between HA-use and sociodemographic and audiological factors in population-based studies of older adults [11, 27]. Factors associated with use of HA in a population-based study of older adults may differ from those among patients in audiological clinics. A large population-based study may be better suited to study multiple factors simultaneously than a clinical study. Thus, the importance of audiological measured low, medium and high frequency HI adjusted for sociodemographic factors may be studied both with and without adjustment for self-perceived severity of hearing loss. A study exploring the relations between use of HA (versus no use) and sociodemographic factors, measured HI and self-perceived severity of hearing loss in a population sample of older adults can give us a better understanding of the driving forces for use of HA in the society.

The study aimed to assess the prevalence of hearing aid use in older adults (65 years and more), and to investigate how socioeconomic and hearing related factors such as degree of HI and being bothered by HI were associated with use of hearing aid.

## Methods

### Study population

**Fig. 1:**
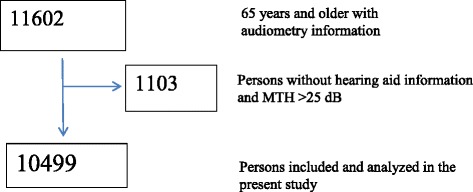
Flowchart for inclusion of participants in the present population-based HUNT2-study

The second wave of the Nord-Trøndelag Health Study took place in 1995–1997 (HUNT2). All inhabitants aged 20 years or older residing in Nord-Trøndelag County were invited to participate. In HUNT 2 93,898 persons were invited, 65,237 participated (69.4 %). HUNT 2 included as an integrated project the Nord–Trøndelag Hearing Loss Study (NTHLS) [29]. The primary aims of the hearing loss study were to assess occurrence, risk factors, and consequences of HI in Norway. Six of the 24 municipalities in the county were not included in the hearing loss study. The present study uses data from persons who were 65 years or older at the HUNT 2 examination time and participated in HUNT 2 and the hearing examination. From 18,763 invited persons in this age group, 11,602 subjects (61.8 %) participated in the NTHLS (see Fig. 1). Owing to missing data on single variables in the material, the number of participants in the analyses varies from 7957 to 10,499.

In many aspects Nord-Trøndelag is considered fairly representative of Norway (geographically, and regarding economy, industry, sources of income, trends in work related disability, age distribution, morbidity and cause-specific mortality), but it has no large cities and a somewhat lower level of education than the national average.

The study has been approved by the Norwegian Regional Committee for Medical Research Ethics.

### Measurements

*Audiometry* in HUNT2*.* Air- conduction hearing thresholds were obtained by pure tone audiometry with Interacoustics AD25 automatic self-administered audiometers with TDH-39 earphones linked to a personal computer (PC). Data were automatically stored at the PC after testing. A few persons were offered a manual audiometry because they were not able to follow the instruction for the automatic procedure. Bone conduction thresholds were not examined and masking was not used. Otoscopy was carried out prior to the audiometry by two professional audiologists in a sub-sample with 6415 subjects. 10.9 % of the ears were observed with abnormal pinna or external ear or with abnormal tympanic membrane and 2.5 % were observed with ear canal obstruction, and the rest were considered normal [30]. These complications were estimated to account for a mean threshold shift from 0.0 dB to 0.3 dB for the various frequencies in the total subsample.

The standard frequencies included in the tests were 250, 500, 1000, 2000, 3000, 4000, 6000, and 8000 Hz. Thresholds were determined in accordance with ISO 8253-1 (1989) [31]. Mean hearing thresholds were defined as: 1) low frequency hearing level (250 Hz and 500 Hz, thresholds averaged over frequencies and both ears); 2) medium frequency hearing level (1000 Hz and 2000 Hz); and 3) high frequency hearing level (3000 Hz, 4000 Hz, 6000 Hz and 8000 Hz). Test-retest reliability were 0.89 for low frequencies, 0.98 for medium frequencies, and 0.99 for high frequencies [29]. In addition, mixed frequency hearing level (500, 1000, 2000 and 4000 kHz, thresholds averaged over frequencies and both ears) was calculated.

The audiometers were calibrated every six month. Semi-portable, dismountable sound attenuation booths were used in rooms specially selected to avoid background noise. Background noise was measured on a random sample and met the recommended standard for test administration (ISO 8253-1, 1989) [31]. Further information of the test procedure is published elsewhere [29].

*Use of HA* was observed by questionnaire. Those who reported that they knew they had a hearing loss, were asked to answer the following question: Do you use hearing aid? (Yes/no.) If responding “no” to having a hearing loss, missing data on hearing aid use were treated as no hearing aid. If confirming to have a hearing loss or not responding to this question, and if the measured mean hearing threshold value was lower than 26 dB, missing data on the hearing aid item were treated as no hearing aid *(n = 599).* Otherwise participants with missing hearing aid data were excluded *(n = 1103).*

*Being bothered by hearing loss* was assessed with one self-report item in HUNT 2, questionnaire 1 (Q1). Those who reported that they knew they had an impaired hearing, were asked how much bothered they were by this impairment, either a little (1), moderately (2), or a lot (3). Subjects who did not report a hearing impairment in Q1 were scored 0.

*Demographic information* includes information about marital status and highest level of education. Marital status gives information of whether the participant has a spouse or not. The highest educational level achieved reported in HUNT 2 (Q1) was scored according to the OECD guidelines for classification [32]. The original individual data were grouped into three classes (1 = up to 10 years education; 2 = vocational or high school education; and 3 = college or university).

### Statistics

Data were analyzed by use of SPSS version 22.0 (IBM SPSS, Chicago, IL, USA).

The main outcome, using HA (versus not), was studied using logistic regression analysis for men and women separately. Preliminary analyses were run entering the independent variables low, medium and high frequency thresholds as categorical variables with every 10 dB as a separate category. The results showed an almost linear effect for medium and high frequency thresholds, that is, a similar increase in odds ratios (OR) for every 10 dB change in hearing threshold along the whole distribution for medium and high frequency thresholds. Thus, medium and high frequency thresholds were entered as continuous variables scaled with 10 dB as units for men and women. A linear increase in OR from 20 dB for every 10 dB change in low frequency hearing threshold was found up till 70 dB, but not thereafter. Other variables of interest for use of hearing aids explored in unadjusted analysis were age, marital status, education and being bothered by hearing loss. Age was in the preliminary analyses found to be linearly associated with the outcome and was entered as a continuous variable in the analyses. In all regression analyses the lowest score was set as reference group whenever possible.

Interaction effects between hearing thresholds and age, marital status and education on hearing aid use were first checked separately in men and women, and so were interaction effects between age, marital status and education. Lastly, interaction effects were studied in the total sample, also entering the interaction terms HI X gender.

In the first models sociodemographic variables and measured hearing thresholds in low, medium and high frequencies were included. In the second model an additional variable, being bothered by hearing loss were included. The results were reported as OR with 95 % confidence intervals (CI). *P*-values ≤ 0.05 were considered statistically significant.

## Results

Off all participants, 62 % (6480 of 10,499) had a mean HI (based on the 0.5, 1, 2, 4 kHz frequencies and both ears) higher than 25 dB and 14 % (1472 of 10,499) used HA. From the latter 889 (60.4 %) were men (see Table 1). The mean and standard deviation of age was higher in women and men using HA than those not using HA (i.e. 77.9; 6.4 years versus 73.4; 6.4 years for women and 75.8; 6.4 years versus 72.8; 5.7 years for men).

**Table 1:** Characteristics of the participants in the population-based HUNT 2 Study by using HA equipment or not

		HA users	Not HA users
Number		1472 (100)	9027 (100)
*Sociodemographic variables*	N (%)		
Men	N (%)	889 (60.39)	3926 (43.49)
Age			
65–69 years	N (%)	258 (17.53)	3114 (34.96)
70–75 years	N (%)	379 (25.74)	2824 (31.28)
75–79 years	N (%)	319 (21.67)	1679 (18.60)
80–84 years	N (%)	278 (18.89)	868 (9.62)
≥ 85 years	N (%)	167 (11.35)	304 (3.37)
Having spouse^a^	N (%)	916 (62.23)	5502 (60.95)
Education^a^			
Up to ten years education	N (%)	840 (57.07)	5413 (59.96)
Vocational and general education	N (%)	315 (21.40)	1757 (19.46)
College and university	N (%)	75 (5.10)	593 (6.57)
*Audiological variables*			
Hearing thresholds			
Low frequency	Mean (SD)	40.61 (17.51)	23.31 (10.96)
Medium frequency	Mean (SD)	53.27 (15.61)	25.81 (13.70)
High frequency	Mean (SD)	74.13 (14.55)	50.27 (19.02)
Bothered by hearing loss^a^			
Not at all	N (%)	97 (6.59)	5635 (62.42)
Yes, a little	N (%)	677 (45.99)	2024 (22.42)
Yes, a lot	N (%)	561 (38.11)	285 (3.16)

^a^Numbers do not sum to 10,499, due to missing information on single independent variables in the material

In the fully adjusted logistic regression model (including sociodemographic variables, measured hearing thresholds in low, medium and high frequencies and being bothered by hearing loss), we found use of hearing aid to be associated with higher education, higher medium frequency hearing thresholds, and being bothered by hearing loss in both men and women. The results are shown in Table 2. Low frequency hearing thresholds were not associated with hearing aid use in women. In men, low frequency hearing thresholds from 30 dB up to 50 dB were associated with increased use of hearing aids, but low frequency hearing thresholds ≥ 70 dB were associated with decreased use of HA. Men living with a spouse had higher odds (OR = 1.90; 95 % CI = 1.35–2.52) for using hearing aids compared to men without a spouse. For women there was no difference (OR = 1.04; 95 % CI = 0.76–1.42) between those living with or without a spouse in their use of hearing aids. Men and women living without a spouse did not differ in their use of HA. The interaction effect between gender and having a spouse was significant (*p* = 0.01). No other interaction test reached significance.

**Table 2:** Unadjusted and adjusted estimates for HA-use (versus not) for women and men by sociodemographic and audiological variables^a^

Women (*n* = 5684)	HA-use	No HA	Unadjusted		Model 1		Model 2	
	(N)	(N)	OR	95 % CI	OR	95 % CI	OR	95 % CI
*Sociodemographic variables*								
Age			**1.124**	**1.109–1.140**	0.994	0.971–1.017	0.996	0.971–1.022
Having spouse^b^								
No	370	2515		Reference		Reference		Reference
Yes	213	2579	**0.561**	**0.470–0.670**	1.064	0.799–1.415	1.035	0.755–1.418
Education^b^								
Up to ten years education	386	3394		Reference		Reference		Reference
Vocational and general education	74	653	0.996	0.766–1.295	**1.489**	**1.042–2.127**	**1.558**	**1.051–2.308**
College and university	19	250	0.668	0.414–1.078	1.239	0.668–2.300	1.108	0.574–2.141
*Audiological variables*								
Low frequency hearing thresholds								
<20 dB	22	1763		Reference		Reference		Reference
20 ≤ HT < 30 dB	64	1986	**2.582**	**1.584–4.210**	0.996	0.572–1.736	0.925	0.494–1.734
30 ≤ HT <40 dB	120	887	**10.841**	**6.833–17.202**	1.468	0.851–2.532	1.291	0.704–2.366
40 ≤ HT <50 dB	150	314	**38.282**	**24.087–60.841**	**2.076**	**1.165–3.697**	1.554	0.819–2.950
50 ≤ HT <60 dB	108	103	**84.026**	**50.982–138.489**	1.765	0.920–3.388	1.324	0.643–2.726
60 ≤ HT <70 dB	58	24	**193.663**	**102.640–365.405**	1.985	0.817–4.826	1.826	0.670–4.905
HT ≥ 70 dB	61	24	**203.680**	**108.227–383.319**	0.845	0.301–2.369	0.876	0.284–2.704
Medium frequency hearing thresholds			**3.595**	**3.288–3.930**	**3.473**	**2.936–4.109**	**2.816**	**2.340–3.390**
High frequency hearing thresholds			**2.441**	**2.280–2.614**	1.020	0.901–1.155	0.962	0.836–1.106
Bothered by hearing loss^b^								
Not at all	45	3374		Reference				Reference
Yes, a little	237	877	**20.262**	**14.607–28.106**			**6.409**	**4.276–9.583**
Yes, a lot	246	131	**140.798**	**97.981–202.326**			**15.298**	**9.607–24.359**
Nagelkerke R Square in % **-2Log likelihood**						54.9 1655.068		61.3 1305.535
** Men** **(** *n* ** = 4815)**								
*Sociodemographic variables*								
Age			**1.087**	**1.074–1.100**	0.986	0.969–1.003	1.004	0.984–1.024
Having spouse^c^								
No	184	1000		Reference		Reference		Reference
Yes	703	2923	**1.307**	**1.095–1.561**	**2.000**	**1.556–2.571**	**1.901**	**1.346–2.517**
Education^c^								
Up to ten years education	454	2019		Reference		Reference		Reference
Vocational and general education	241	1104	0.971	0.817–1.154	**1.457**	**1.166–1.820**	**1.384**	**1.086–1.764**
College and university	56	343	0.726	0.538–0.980	1.396	0.954–2.047	1.482	0.985–2.230
*Audiological variables*								
Low frequency hearing thresholds								
<20 dB	116	1879		Reference		Reference		Reference
20 ≤ HT < 30 dB	212	1352	**2.540**	**2.004–3.219**	1.136	0.854–1.511	1.089	0.799–1.485
30 ≤ HT <40 dB	234	471	**8.048**	**6.303–10.276**	**1.710**	**1.244–2.349**	**1.462**	**1.034–2.067**
40 ≤ HT <50 dB	165	152	**17.584**	**13.166–23.484**	**2.180**	**1.482–3.208**	**1.887**	**1.240–2.870**
50 ≤ HT <60 dB	86	42	**33.168**	**21.927–50.172**	**2.137**	**1.213–3.764**	1.767	0.952–3.282
60 ≤ HT <70 dB	41	10	**66.413**	**32.448–135.929**	**3.481**	**1.328–9.1263**	2.698	0.957–7.613
HT ≥ 70 dB	35	20	**28.347**	**15.863–50.655**	**0.281**	**0.118–0.666**	**0.299**	**0.119–0.751**
Medium frequency hearing thresholds			**2.801**	**2.617–2.997**	**2.720**	**2.438–3.034**	**2.274**	**2.019–2.560**
High frequency hearing thresholds			**1.960**	**1.855–2.072**	1.011	0.928–1.102	0.925	0.840–1.018
Bothered by hearing loss^c^								
Not at all	52	2261		Reference				Reference
Yes, a little	440	1147	**16.680**	**12.405–22.427**			**7.458**	**5.225–10.644**
Yes, a lot	315	154	**88.938**	**63.574–124.420**			**17.024**	**11.238–25.778**
Nagelkerke R Square in % **-2Log likelihood**						47.2 2518.553		54.4 2062.380

The variables presented in the models are adjusted for each other. Model 1included socio-demographic variables and measured hearing thresholds in low, medium and high frequencies and in Model 2 included additional being bothered by hearing loss

*OR* odds ratio, *CI* confidence intervals

^a^Medium and high frequency hearing thresholds were entered as continuous variables scaled with 10 dB as units for men and women

^b^Numbers do not sum to 5684, due to missing information on single independent variables in the material for women

^c^Numbers do not sum to 4815, due to missing information on single independent variables in the material for men

The number of participants in analyses vary owing to missing information on single independent variables,

-participating women: 5684 in unadjusted analysis without missing, 4769 in model 1 and 4116 in model 2,

-participating men: 4815 in unadjusted analysis without missing, 4212 in model 1 and 3841 in model 2

Bold numbers in the table are significant associations

## Discussion

To the best of our knowledge, this is the first study which has assessed association between sociodemographic and hearing related variables - including measured HI and self-reported hearing loss - and use of HA in a large population-based sample of older persons. In all, less than one seventh (14 %) of the participants used HA, but about two third had a HI (based on 0.5,1,2,4 kHz) higher than 25 dB over both ears. Use of HA was associated with higher degree of HI, being bothered by hearing loss and education above ground level in both men and women. Men without a spouse were less likely to use HA compared to men with a spouse. Women without spouse were not more or less likely to use HA than women with spouse or men without a spouse.

The prevalence of HA-use among community-dwelling older adults was in the present study equally low as other population-based studies (8–13 %) [9, 10, 12]. Thus, few older adults use HA in everyday life, even if the prevalence of HI in older adults is quite high [1–4], and HA may enable more efficient use of the person’s remaining hearing through an increase in speech perception and recognition of non-verbal sounds [5].

It is expected that the need to improve hearing with use of HA increases as the degree of HI worsens. Even so, two review reports indicate that the evidence of an association between higher degree of HI and increasing chance of using HA after an audiological HA fitting process is mixed [22, 24]. In population-based studies HI may be estimated audiometrically or with use of self-reported questionnaire information about hearing acuity in various situations. Applied on the whole population, the former is quite resource demanding, whereas the latter is less reliable [33, 34]. We identified two population-based studies with a relatively limited sample size (N < 3000) which assessed the association between degree of measured HI and either HA-use or a 5 year incidence of HA-use as outcome. These studies have reported mean HI (based on the 0.5, 1, 2, 4 kHz frequencies) higher than 25 dB bilaterally associated with use of HA [12, 27]. In the present study with a larger sample size we were able to study the dose-response relationship along the whole distribution of hearing loss and found a linear relationship for medium frequency and a monotonous relation up till 70 dB for low frequency hearing. Unlike previous studies we were also able to examine the importance of low, medium and high frequencies for use of HA independently. No independent effect was found of high-frequency hearing levels for HA-use. For both women and men a 10 dB increase in mid –frequency hearing increased the odds for HA-use more than twofold. In fully adjusted analysis of men, higher low-frequency HI from 30 up to 50 dB increased the odds for HA-use, but when the low-frequency threshold was 70 dB or higher the odds for HA-use was even lower than for the reference group with low-frequency threshold less than 20 dB. When hearing is gradually lost, low-frequency and mid-frequency hearing is usually kept longer intact than high-frequency hearing and the low - frequency hearing is retained the longest. Thus, the results probably reflect that HA-use starts when HI involves the medium frequencies band, rather than the high frequency band. When the low frequency HI is 70 dB or higher the effect of HA-use may be limited, especially if the medium and high frequencies are even poorer.

Furthermore, as expected and previously found in clinical audiological studies and population-based studies, experienced consequences of HI were positively associated with HA-use [12, 22, 27], i.e. older adults being bothered by hearing loss were much more likely to use HA compared to those not bothered by hearing loss, also after adjustment for sociodemographic conditions and HI over three frequency bands. In addition, HA-use was in the same analysis not associated with increasing age. This finding is in line with the majorities of previous studies [9, 22, 24], but not all [12, 27]. Moreover, those having vocational and general education were more likely to use HA compared to those with lower education. This may indicate an undesirable pro-educated use of HA. Vikum et al have accordingly shown that people with high socioeconomic status use specialist health services more frequently than people with low socioeconomic status in the same area [35]. It is left to future studies to decide whether education is associated with help seeking behavior, access to treatment, or HA-use after fitting, however. Degree of income, which is related to level of education, has been found to be positively associated with HA provision but of less importance when treatment and HA is funded by the authorities [24]. Also, financial factors like costs of batteries and repairs have been found of importance for no use of HA after HA were fitted [36].

This large scale population study made it possible to study interaction between independent variables for HA-use, and we found an interaction between gender and marital status. Men having a spouse were twice as likely to be HA-users compared to men without a spouse, but there were no difference in use of HA between women with and without a spouse. We did not study the participants’ communication demands or the role of significant others in the present study, i.e. the spouse of the participants or other close relatives. However, others have found the communication demands and the role of significant others to influence audiological help seeking and/or HA-use [24, 36]. Women regularly have a higher pitch when speaking compared to men. Thus, it may be harder or more demanding for a man with HI without HA to communicate with his wife, than for women to communicate with their husband without HA even if their HI were about the same.

The strengths of this study are mainly the large study sample (10,499 persons aged 65 years or more) and the objective measure of HI. The participants were aware of this study's specific hypotheses during data collection, which limits the risk of reporting bias of HA-use.

The limitations of the present study need to be addressed. Even if the effects of the various audiological variables were adjusted by sociodemographic variables, we cannot rule out that there are confounders which we have not adjusted adequately for. In addition to communication demands and role of significant others, which we did not have information about, personality and cosmetic demands may influence HA acquiring and later HA-use [22, 24, 37]. Secondly, due to the epidemiological nature of the study, otoscopy was not carried out prior to the audiometry for the full sample. Previous results from otoscopy in a subsample, showed that non-normal otoscopy findings in 13.3 % of the subsample, only accounted for a mean threshold shift of 0.0 dB to 0.3 dB in this subsample, implying that missing otoscopy data have hardly affected our results much [30]. Detailed information about previous clinical audiological testing and HA acquiring is missing, therefore it was impossible to assess the proportion of persons who have acquired HA but do not use it and also impossible to explore factors associated with acquiring HA in the present study [27]. Thirdly, we do not have information about whether the HA-users were part-time or fulltime users. Even so, earlier studies have shown that self-reported use of HA correlates highly with objective measures of use [38].

There is usually some uncertainty related to the generalizability of results from population studies like ours. The hearing examination represented only a small part of the total examination program in the HUNT 2 study, so there is little reason to suspect that hearing acuity has substantially influenced the recruitment to the study. A reduction of the study sample due to missing data on single questionnaire items may have affected the estimated occurrence of HA-use somewhat, but has hardly more than trivially affected the observed relationships between the predictors and the outcome. Nord-Trøndelag county is considered to be quite representative of Norway, so there is also little reason to doubt that these results are valid nationwide. The extent to which the results can be generalized internationally is more questionable. It seems reasonable to assume that the results are essentially valid at least for rather wealthy societies with refund arrangements for HA similar to the Norwegian. A more important limitation to the generalizability is the age of the data, especially because the HA technology has developed considerably since 1998. Statistics on number of acquired HA per year have more than doubled in Norway since the time of our data collection, from 39 778 in 1998 to 84 606 in 2015 (http://www.n-t-a-f.org/HA-statistikk.htm). That does not necessarily show that the real HA use has been doubled, since we have a higher HI prevalence in the nation due to the ageing of the population, people may change HAs more frequently today (normally every sixth year) than twenty years ago and that it is more usual to choose HA for both ears now than 20 years ago, but it may reflect a higher proportion of HA users. But even if better technology may have caused a somewhat increased occurrence of HA-use during the last two decades, most of the reasons for using or not using HA have hardly changed fundamentally. Unfortunately, absence of more recent audiometrical data connected to use of HA makes it difficult to know the extent to which our results are valid for today’s hearing aid use.

## Conclusion

The proportion of HA-users, 14 % among people aged 65 years or older, was low considering the much larger proportion with HI. Use of HA was strongly associated with higher degree of HI along most of the distribution of HI. Being bothered by hearing loss was a very strong indicator of HA-use even after adjusting for measured HI. Education above ground level also was asociated with HA-use in both men and women. Men without a spouse were less likely to use HA compared to men with a spouse, whereas HA-use in women did not depend on marital status.

## Abbreviations

CI, confidence intervals; dB, decibel; HA, hearing aid; HI, hearing impairment; kHz, kilo Hertz; OR, odds ratio
